# Emerging ultra-narrow-band cyan-emitting phosphor for white LEDs with enhanced color rendition

**DOI:** 10.1038/s41377-019-0148-8

**Published:** 2019-04-10

**Authors:** Ming Zhao, Hongxu Liao, Maxim S. Molokeev, Yayun Zhou, Qinyuan Zhang, Quanlin Liu, Zhiguo Xia

**Affiliations:** 1 The Beijing Municipal Key Laboratory of New Energy Materials and Technologies, School of Materials Sciences and Engineering, University of Science and Technology Beijing, 100083 Beijing, China; 2 Laboratory of Crystal Physics, Kirensky Institute of Physics, Federal Research Center KSC SB RAS, Krasnoyarsk,, 660036 Russia; 3 Siberian Federal University, Krasnoyarsk, 660041 Russia; 4 Department of Physics, Far Eastern State Transport University, Khabarovsk, 680021 Russia; 5 State Key Laboratory of Luminescent Materials and Devices and Guangdong Provincial Key Laboratory of Fiber Laser Materials and Applied Techniques, South China University of Technology, 510641 Guangzhou, China

**Keywords:** Inorganic LEDs, Fluorescence spectroscopy

## Abstract

Phosphor-converted white LEDs rely on combining a blue-emitting InGaN chip with yellow and red-emitting luminescent materials. The discovery of cyan-emitting (470–500 nm) phosphors is a challenge to compensate for the spectral gap and produce full-spectrum white light. Na_0.5_K_0.5_Li_3_SiO_4_:Eu^2+^ (NKLSO:Eu^2+^) phosphor was developed with impressive properties, providing cyan emission at 486 nm with a narrow full width at half maximum (FWHM) of only 20.7 nm, and good thermal stability with an integrated emission loss of only 7% at 150 °C. The ultra-narrow-band cyan emission results from the high-symmetry cation sites, leading to almost ideal cubic coordination for UCr_4_C_4_-type compounds. NKLSO:Eu^2+^ phosphor allows the valley between the blue and yellow emission peaks in the white LED device to be filled, and the color-rendering index can be enhanced from 86 to 95.2, suggesting great applications in full-spectrum white LEDs.

## Lighting: Shining in the cyan valley

Luminescent crystals that efficiently emit light in the narrow cyan color wavelength range, from 470 to 500 nanometers, could be used to fill a gap or “valley” in light-emitting diodes (LEDs) intended to mimic the full spectrum of daylight. There is great interest in developing more efficient and cost-effective full daylight spectrum LED lighting sources. These can create more natural indoor lighting conditions that are also believed to be more beneficial for health. Researchers in China and Russia, led by Zhiguo Xia at the University of Science and Technology Beijing, developed a suitable inorganic crystalline compound showing narrow cyan emission, with the formula Na_0.5_K_0.5_Li_3_SiO_4_:Eu^2+^. The many possible applications include LEDs for daylight spectrum lamps used to treat the low mood and depression associated with Seasonal Affective Disorder (SAD).

## Introduction

White light-emitting diodes (LEDs) have been successfully used in solid-state lighting or backlight units for liquid crystal displays due to their high efficiency, tunable color, durability, long lifetime, energy saving, and environmental friendliness^1–3^. Currently, most commercial phosphor-converted LEDs (*pc*-LEDs) can be achieved by the combination of a blue InGaN chip with yellow-emitting Y_3_Al_5_O_12_:Ce^3+^ (YAG:Ce) phosphor^4,5^. It is acknowledged that such white LEDs have some drawbacks, such as a low color-rendering index (CRI, *R*_a_ < 80) and high correlated color temperature (CCT) (CCT > 4000 K), owing to the lack of the red region of the spectrum^6^. After the discovery of highly efficient red-emitting phosphors, such as CaAlSiN_3_:Eu^2+^, Sr_2_Si_5_N_8_:Eu^2+^, K_2_SiF_6_:Mn^4+^, or SrLiAl_3_N_4_:Eu^2+^, the *R*_a_ values can be enhanced to ~90^7–11^. Moreover, some solid solution phosphors with multiple emission centers have been discovered to improve the *R*_a_ values^11,12^. However, there is still a challenge in enhanced color rendition because of the cyan gap between blue and yellow emission in the 470–500 nm region, which is not suitable for high-quality general lighting. Hence, it is of fundamental importance to develop a novel phosphor emitting in this spectral region for filling the valley between the blue and yellow emission peaks in a white LED device. Accordingly, the development of narrow-band cyan-emitting phosphors excited by blue light with a small Stokes shift is necessary for improving the optical performance of pc-LEDs^13^.

The design of narrow-band cyan-emitting phosphors plays a significant role in enhancing the color rendition to compensate for the peak valley between the blue and yellow emission peaks. However, luminous efficacy and color rendition are generally in a trade-off relationship^14,15^. Thus, the narrow-band characteristic will decrease the opportunity for spectral overlapping of the excitation spectrum of YAG:Ce and emission spectrum of the cyan-emitting phosphor, which avoids the reabsorption effect to realize the maximum achievable luminous efficacy^4^. Presently, there are few narrow-band cyan-emitting phosphors that can be excited by blue light. The typical example is BaSi_2_O_2_N_2_: Eu^2+^, which possesses an emission band with the peak at ~495 nm (FWHM = ~32 nm)^16^. However, it has poor chemical and thermal stability due to its layered crystal structure. Recently, a new narrow-band cyan-emitting oxonitridoberyllate phosphor Sr[Be_6_ON_4_]:Eu^2+^ (*λ*_em_ = 495 nm, FWHM = 35 nm) has been reported^13^. Nevertheless, the harsh synthetic conditions and toxicity of this phosphor are serious drawbacks for its application. Therefore, novel stable and nontoxic phosphors with narrow-band cyan emission are extremely required for application in enhancing the color rendition of *pc*-LEDs.

Recently, the development of narrow-emitting phosphors has been continuously pursued for versatile applications in solid-state lighting or backlight units for liquid crystal displays, and understanding structure-property relations for the creation of narrow-band emission is also a great challenge^17^. The mineral-inspired prototype evolution and new phase construction proposed by our group have demonstrated great potential in discovering new phosphors for emerging applications^5,18^. For example, the newly reported narrow-band materials, Be-containing phosphors, SrLi_2_[Be_4_O_6_] is related to the BaLi_2_[(Al_2_Si_2_)N_6_] prototype, and Sr[BeSi_2_N_4_] originates from the Sr[Be_3_O_4_] model^19–21^. Moreover, narrow-band nitride phosphors are extensively investigated in the UCr_4_C_4_-type model, and one of the typical examples is red-emitting Sr[LiAl_3_N_4_]:Eu^2+^ with a highly condensed, rigid network, and highly symmetric dopant sites^10^. Therefore, oxide-based UCr_4_C_4_-type compounds with narrow-band emission can also be expected^22–24^. Inspired by these findings, an emerging ultra-narrow-band cyan-emitting phosphor in silicate materials was demonstrated in this work, and we adopted structural modeling in this system to design a novel narrow-band cyan-emitting silicate phosphor. Accordingly, the totally new [Na_0.5_K_0.5_][Li_3_Si]O_4_ phase, originating from the UCr_4_C_4_-type compound NaLi_3_SiO_4_, was discovered in this host family. The relationship between the ultra-narrow-band cyan emission and three different cations with high-symmetry cation sites leading to almost ideal cubic coordination was analyzed. Eu^2+^-doped [Na_0.5_K_0.5_][Li_3_Si]O_4_ (abbreviated as NKLSO:Eu^2+^) phosphor exhibits an ultra-narrow-band emission with a peak at 486 nm, FWHM = 20.7 nm and a Stokes shift = 1069 cm^−1^. Compared to that of the red-emitting Sr[LiAl_3_N_4_]:Eu^2+^ phosphor, the emission peak of NKLSO:Eu^2+^ has a significant blueshift. Because the formation energy of N^3-^ from atomic N ( + 2300 kJ mol^−1^) is higher than that of O^2−^ from atomic O ( + 700 kJ mol^−1^), the bonding in nitrides is notably more covalent than that in oxides^25^. Therefore, the 5*d* energy levels of Eu^2+^ in nitride would be lower compared to that in oxide, which results in the lower 5*d* −4*f* transition energy of the Sr[LiAl_3_N_4_]:Eu^2+^ nitride phosphor than that of the NKLSO:Eu^2+^ oxide phosphor. Moreover, this NKLSO:Eu^2+^ phosphor possesses good thermal stability with an integrated emission intensity of 93% at 150 °C. By combining the cyan phosphor NKLSO:8%Eu^2+^, the commercial yellow phosphor YAG:Ce and the commercial red phosphor K_2_SiF_6_:Mn^4+^(KSF:Mn^4+^) with a blue LED chip, a white LED with a high *R*_a_ of 95.2 was acquired, which demonstrated that the phosphor can cover the cyan gap to enhance the CRI in the as-fabricated white LED.

## Results

### Crystal structure and morphology of NKLSO

**Fig. 1:**
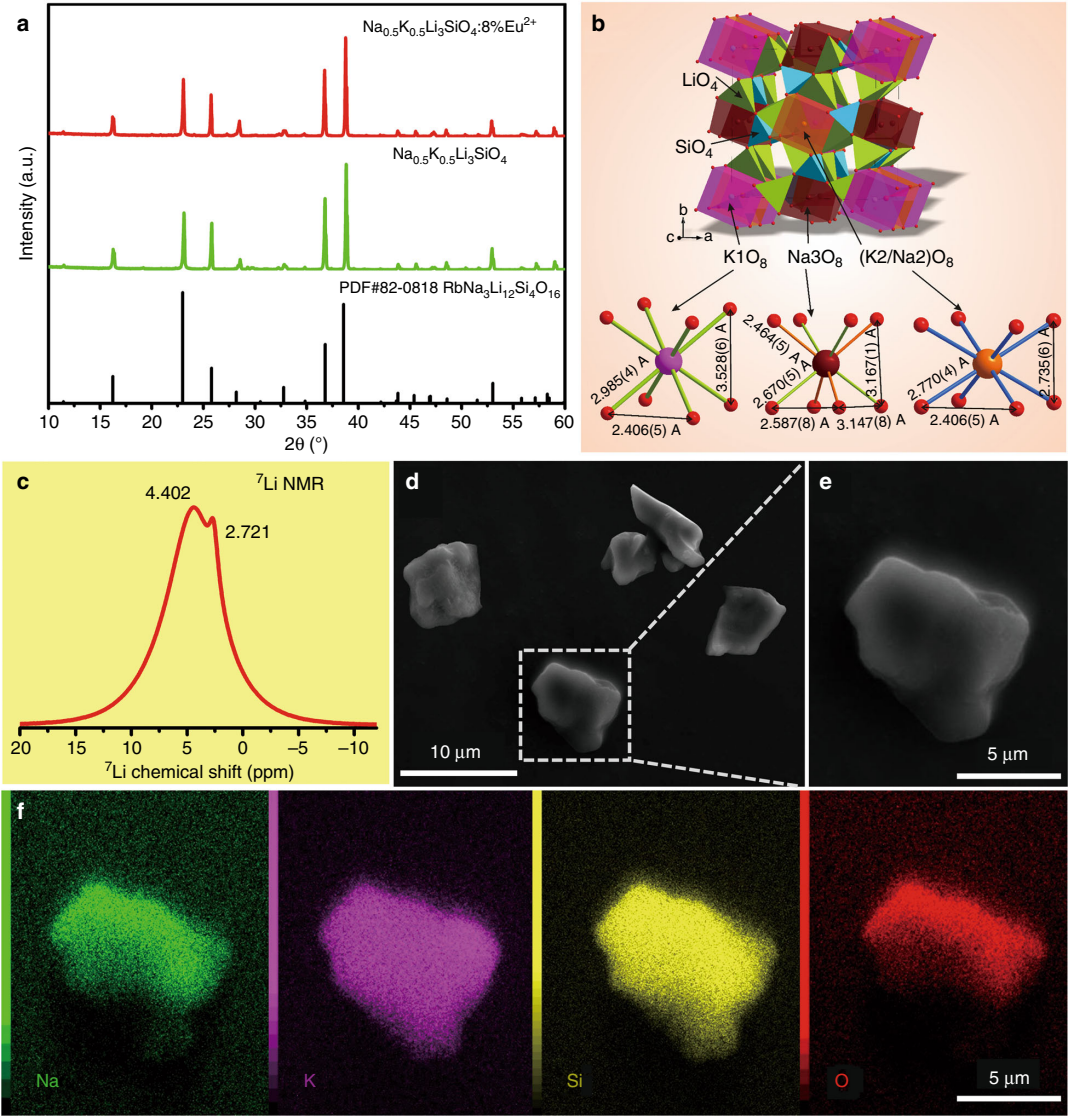
Structural characterization, morphology and composition of NKLSO:Eu^2+^ phosphor. **a** XRD patterns of NKLSO and NKLSO:8%Eu^2+^ and standard pattern of PDF card No.82-0818 as reference. **b** Crystal structure of NKLSO and the coordination spheres of K1, Na3, and K2/Na2. **c** ^7^Li solid-state NMR spectrum of NKLSO. **d** SEM image of NKLSO microcrystal particles and **e** an enlarged particle. **f** EDS elemental mapping images of selected NKLSO particles

Figure 1a shows the XRD patterns of NKLSO and NKLSO:8%Eu^2+^, and all the diffraction peaks can be indexed to the reported pattern of RbNa_3_Li_8_(Li(SiO_4_))_4_ (PDF card No.82-0818)^26^, which indicates that NKLSO is isostructural to it. Hence, the crystal structure of RbNa_3_Li_8_(Li(SiO_4_))_4_^26^ is taken as the starting model for the Rietveld refinement of NKLSO. Three cation sites (Rb, Na1, and Na2) exist in the host, and all of them are occupied by K/Na mixed ions. The ratios are allowed to refine with restriction that the sum of occupations occ(Na) + occ(K) is equal to 1 at all sites. After preliminary refinement, it is found that the first cation is fully occupied by K^+^ ions, the second site is intermixed K/Na with a ratio of 0.67/0.33, and the third site is fully occupied by Na^+^ ions (Fig. 1b). These occupations were accounted for in the new model, and K/Na ratios were fixed in the first and third sites. The thermal parameters of all ions were refined isotropically. The as-obtained structural parameters were stable with low *R*-factors (Supplementary Fig. S1a and Supplementary Table S1). NKLSO was found to crystallize in a tetragonal crystal system with space group *I*4/*m*, and its unit cell parameters are *a* = *b* = 10.9447 (1) Å and *c* = 6.26244 (8) Å. Coordinates of atoms and main bond lengths are shown in Supplementary Table S2 and Supplementary Table S3, respectively. The chemical formula from refinement can be written as Na_0.58(4)_K_0.42(4)_Li_3_SiO_4_, which is close to the suggested formula within 2σ. The crystallographic information file of NKLSO is also provided in the Supplementary Information.

Regarding the crystal structure (Fig. 1b and Supplementary Fig. S1b), LiO_4_ and SiO_4_ tetrahedra connected to each other by corner- and edge-sharing to form a highly condensed three-dimensional framework with the degree of condensation *κ* = 1 that is equal to the atomic ratio (Li, Si): O, and three different cations, K1, K2/Na2, and Na3, are filled in vierer ring channels along [001]. All of these ions are coordinated with eight O^2−^ ions forming cubic polyhedrons with high-symmetry characteristics (Fig. 1b). For example, the high-symmetry 4/*m* of the K1 and K2/Na2 sites lead to almost ideal cubic coordination with equal *d*_(Na,K–O)_ bond lengths and with a difference between some *d*_(O–O)_ only (Fig. 1b), which strongly contribute to the as-observed narrow emission bands, as discussed below. The Na3 site has a relatively low symmetry of –4, and the bond lengths *d*_(Na3–O)_ divide into two groups, leading to distorted cubic coordination. The alkali metal ions replaced by Eu^2+^ would lead to an increase in positive charge of the unit cell; hence, the charge compensation can be associated with the formation of vacancies in cations instead of anions, such as 2Na^+^ → Eu^2+^ + V_Na_, 2 K^+^ → Eu^2+^ + V_K_, Li^+^ + K^+^ → Eu^2+^ + V_Li_, and Li^+^ + Na^+^ → Eu^2+^ + V_Li_. The numerous different combinations prevent reliable determination of the mechanism, and therefore, we do not speculate about it. However, these substitutions and as-formed vacancies will not have a strong influence on the Eu^2+^ cuboid-like coordination with the relatively stable first coordination sphere of O^2−^ ions.

The local structure, chemical composition and morphology of NKLSO:Eu^2+^ phosphor were further investigated to assess this newly discovered ultra-narrow-band cyan-emitting phosphor. First, the ^7^Li solid-state nuclear magnetic resonance (NMR) spectrum was measured. As displayed in Fig. 1c, the ^7^Li NMR spectrum shows two signals at 2.721 and 4.402 ppm, which are consistent with the two different Li crystallographic sites in this host. Scanning electron microscope (SEM) images of the NKLSO powder sample are depicted in Fig. 1d, e, which indicates that the average particle size of the sample is ~5–10 μm, and a well-developed crystalline particle and smooth surface will help the luminescence properties and incorporation into LED packages. The elemental mapping images (Fig. 1f) show a uniform distribution of Na, K, Si, and O in the particle, and the average atomic ratios Na(0.5):K(0.44):Si(1.1) determined by energy dispersive X-ray spectroscopy (EDS) are consistent with the formula Na_0.58(4)_K_0.42(4)_Li_3_SiO_4_ obtained from the Rietveld refinement result from the XRD pattern.

### Photoluminescence properties

**Fig. 2:**
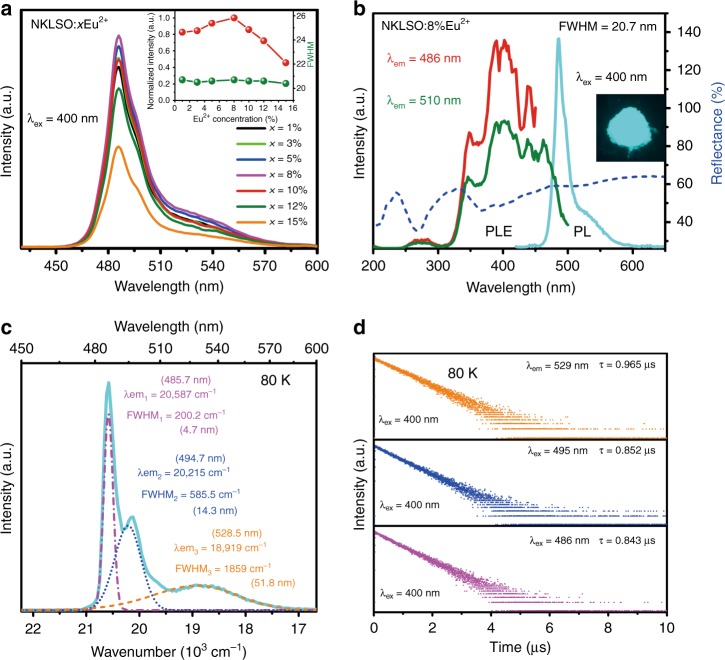
Photoluminescence properties of NKLSO:Eu^2+^. **a** Emission spectra of NKLSO:*x*Eu^2+^ (*x* = 1–15%). The inset shows the dependence of normalized integrated emission intensities and FWHM values on the Eu^2+^ doping concentration. **b** Diffuse reflectance, photoluminescence and photoluminescence excitation spectra of NKLSO:8%Eu^2+^ at room temperature. The inset shows a digital photograph of the cyan NKLSO:8%Eu^2+^ phosphor under a 365 nm UV lamp. **c** Emission spectrum (cyan line) of NKLSO:8%Eu^2+^ at 80 K, and the Gaussian peaks fitting the emission spectrum of this sample. **d** The decay curves at 80 K of NKLSO:8%Eu^2+^ under excitation at 400 nm monitored at different wavelengths

Figure 2a gives the photoluminescence (PL) spectra of NKLSO:*x*Eu^2+^ (*x* = 1–15%) at room temperature (RT), and the optimum Eu^2+^ concentration is determined to be 8 mol%. The diffuse reflectance spectra of NKLSO:*x*Eu^2+^ phosphors are demonstrated in Supplementary Fig. S2, and the absorption intensities increase gradually with increasing Eu^2+^ concentration. Fig. 2b shows the diffuse reflectance, photoluminescence excitation (PLE) and PL spectra at RT of NKLSO:8%Eu^2+^ phosphor. The PLE spectra monitored at two different wavelengths, 486 and 510 nm, show a broad band from 300 to 500 nm, indicating that the phosphor can be excited by ultraviolet light to blue light and is suitable for excitation of the commercial blue chip. The diffuse reflectance spectrum gives a broad absorption band in the range of 330–470 nm, which matched well with the PLE spectrum. The PL spectrum of the NKLSO:8%Eu^2+^ phosphor consists of a dominant asymmetric narrow-band peak at 486 nm (FWHM = 20 nm) and a subordinate shoulder peak at 530 nm under 400 nm excitation, indicating that other luminescence centers may exist, which will be discussed later. In addition, we measured the PL spectra of NKLSO:8%Eu^2+^ under different excitation wavelengths. As shown in Supplementary Fig. S3a, compared to that of the dominant peak at 486 nm, the intensity of the minor peak at 530 nm increased obviously with increasing excitation wavelength, which further proves the existence of other luminescence centers and different evolution behaviors. We calculated the Commission Internationale de L’Eclairage (CIE) chromaticity coordinates and the color purity of the PL spectra of NKLSO:8%Eu^2+^ under 400 nm and 460 nm excitation. The CIE chromaticity coordinates were calculated to be (0.1237, 0.4098) and (0.1430, 0.4380), respectively (Supplementary Fig. S3b), and the color purities were determined to be 68.7% and 66.8%. Under the excitation of 400 nm and 460 nm, the color coordinates of the phosphor moved slightly but still exhibited cyan emission, and the color purities remained almost unchanged. Moreover, the FWHM values of NKLSO:*x*Eu^2+^ remained basically unchanged for different Eu^2+^ doping concentrations (Fig. 2a). The inset image in Fig. 2b shows that the sample has cyan light under 365 nm lamp irradiation. The highly condensed and rigid network results in the narrow-band emission and the small Stokes shift, which is determined as the energy difference between the maximum of the (lowest) excitation band and that of the emission band^27^. The extremely small Stokes shift (1069 cm^−1^) of NKLSO:8%Eu^2+^ also accounts for the confinement in the local structure relaxation of Eu^2+^ in its excited state. Under 400 nm excitation, the RT internal/external quantum efficiencies of NKLSO:8%Eu^2+^ are determined to be 76%/30%, respectively, and the measurement details are shown in Supplementary Fig. S4.

To further verify the correlation between the existing luminescent centers and the crystallographic sites in the crystal structure, the PL spectrum of NKLSO:8%Eu^2+^ at 80 K was measured. It is commonly known that fine emission spectra can be observed at low temperatures owing to decreased thermal broadening. As shown in Fig. 2c, three emission bands located at 486 nm, 495 nm, and 529 nm are clearly observed, which can be ascribed to the existence of three luminescence centers originating from three cation sites occupied by Eu^2+^, as elucidated in the crystal structure analysis. Furthermore, the PL spectrum at 80 K can be divided into three Gaussian peaks (Fig. 2c). Two narrow peaks with FWHM~4.7/14.3 nm can be associated with the high cubic symmetry sites, K1 and K2/Na2 sites occupied by Eu^2+^, and the slightly broad emission band with FWHM~51.8 nm can be associated with Na3 sites occupied by Eu^2+^ owing to the distorted cubic sites. Since we could not refine Eu occupancies in different cation sites using Rietveld refinement due to the very low Eu concentration, the well-known empirical equation given by Van Uitert can be used to further understand the origin of the three emission bands, which provides a good fit to the emission peak for Eu^2+^ discussed hereafter^28^.

1 $$E = Q\left[ {1 - \left( {\frac{V}{4}} \right)^{\frac{1}{V}}10^{ - \frac{{n \times {\rm{EA}} \times r}}{{80}}}} \right]$$

where *E* denotes the position of the *d*-band edge in energy (cm^−1^), *Q* represents the position in energy for the lower *d*-band edge of the free ions, *Q* is 34,000 cm−1 for Eu^2+^ and 50,000 cm^−1^ for Ce^3+^, *V* is the valence of the active ion (here, *V* = 2), *n* is the number of anions in the immediate shell around, *EA* is the electron affinity of the atoms that form anions, and *r* is the radius of the host cation replaced by the active cation. Here, *n* = 8 for the K1, K2/Na2, and Na3 sites, *EA* should have the same values for K1, K2/Na2, and Na3 in this host, and *r* (K1) > *r* (K2/Na2) > *r* (Na3). From the equation, we can see that the larger the value of *r* is, the larger the value of *E*, and the smaller the emission wavelength. Hence, the first peak (486 nm) in the PL spectrum can be assigned to K1 sites, the second peak (495 nm) can be assigned to K2/Na2 sites, and the third broad peak (529 nm) can be assigned to Na3 sites, which is consistent with the previous analysis results depending on different symmetries. Furthermore, the decay behaviors of Eu^2+^ at various sites are generally different depending on the variable chemical environment. To further verify the existence of three Eu^2+^ emissions in NKLSO:Eu^2+^, the decay curves at 80 K monitored at 486, 495, and 529 nm under 400 nm excitation are measured (Fig. 2d). The decay times are determined to be 0.843, 0.852, and 0.965 μs for the peaks at 486, 495, and 529 nm, respectively. The different lifetimes demonstrated that the emissions arise from Eu^2+^ in different lattice sites, and the close decay times (0.843 and 0.852 μs) are ascribed to the quite similar 4 /*m* sites (K1, K2/Na) in the lattice, while the value of 0.965 μs should be ascribed to Eu^2+^ at the different Na3 sites with a relatively low symmetry of –4.

### Thermal quenching properties

**Fig. 3:**
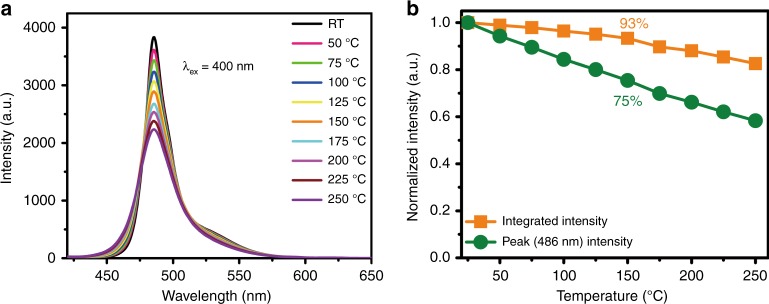
Thermal quenching behavior of NKLSO:8%Eu^2+^ phosphor. **a** Temperature-dependent emission spectra of NKLSO:8%Eu^2+^ phosphor under 400 nm excitation in the temperature range RT–250 °C with a temperature interval of 25 °C. **b** Temperature-dependent normalized integrated emission intensities and normalized peak (486 nm) intensities of NKLSO:8%Eu^2+^

Thermal stability acts as a vital parameter for LED phosphors in practical applications since the LED chip normally reaches temperatures up to ~150 °C at high power. The thermal quenching behavior can be elucidated using the configurational coordinate diagram. Based on this model, the excited luminescent center is thermally activated through phonon interaction and then released through the crossing point between the excited and ground states^29^. Hence, thermal quenching is related to the difference between the equilibrium distance of the ground and excited state (ΔR) in the configurational coordinate diagram, which determines the location of the crossing point^30^. Thus, if the value of ΔR is small, the Stokes shift will be small^27^. Generally, a phosphor with a smaller Stokes shift has stronger structural rigidity^31^, which could significantly reduce the emission loss with increasing temperature. The temperature-dependent emission spectra of NKLSO:8%Eu^2+^ phosphor in the temperature range from RT to 250 °C are shown in Fig. 3a. At 150 °C, NKLSO:8%Eu^2+^ exhibits an integrated emission loss of only 7%, and the emission intensity of the peak (486 nm) is decreased by 25% of the initial intensity, indicating good thermal stability of NKLSO:Eu^2+^ (Fig. 3b). And the variation between the peak intensity and the integrated intensity can be attributed to the broadening effect of the emission band with increasing temperature.

### Performance of as-fabricated white LEDs for enhancing color rendition

**Fig. 4:**
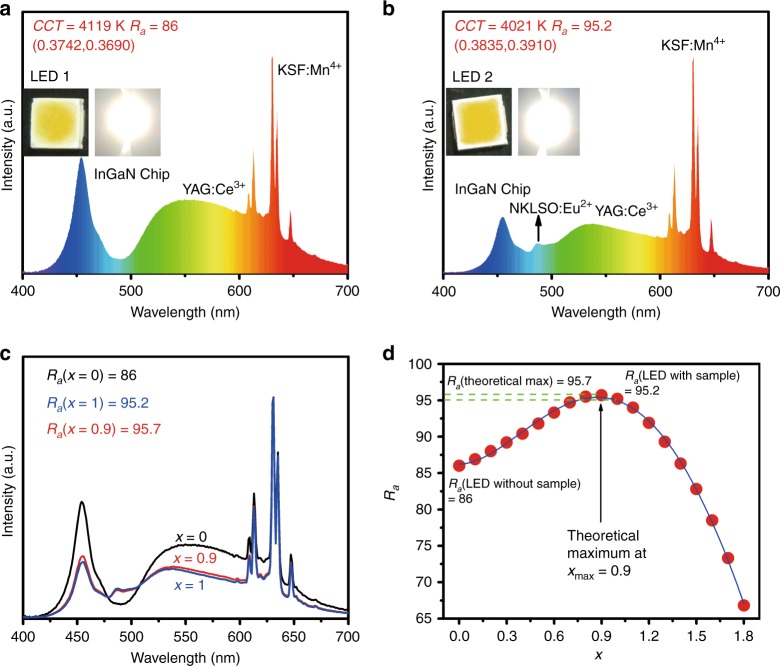
Performance of as-fabricated white LED devices. Emission spectra of the white LED devices fabricated with the commercial yellow phosphor YAG:Ce, the commercial red phosphor KSF:Mn^4+^ and **a** without or **b** with the cyan phosphor NKLSO:8%Eu^2+^ on a blue LED InGaN chip (*λ* = 455 nm) under a current of 20 mA. The insets show photographs of the fabricated white LEDs. **c** Experimental spectra of the white LED without the sample (*x* = 0), white LED with the sample (*x* = 1) and theoretical spectrum *x* = 0.9. **d** The *R*_a_ index plot per *x* for different theoretical spectra, THEOR(λ) = LED1(λ) + *x* × DIFF(λ) with a maximum at *x* = 0.9 and theoretical maximum *R*_a_ (theoretical max) = 95.7

To further evaluate the potential application of NKLSO:Eu^2+^ in white LED lighting for improving the color-rendering index, we fabricated white LED lamps by using the cyan phosphor NKLSO:8%Eu^2+^, the commercial yellow phosphor YAG:Ce and the commercial red phosphor KSF:Mn^4+^ on a blue LED InGaN chip (*λ* = 455 nm) under various drive currents and compared them with the white LED without the cyan phosphor NKLSO:Eu^2+^. Figure 4a, b comparatively show the emission spectra of the as-fabricated LED1 (without the sample) and LED2 (with the sample) devices under a current of 20 mA, and the insets show the photographs of the fabricated and lit white LEDs. LED1 presents warm white light with a CCT of 4119 K, a *R*_a_ of 86 and chromaticity coordinates of (0.3742, 0.3690). However, LED1 shows a cyan gap causing the limited enhancement in the color-rendering index (*R*_a_), which is difficult to exceed 90. As the cyan phosphor NKLSO:8%Eu^2+^ was added into the device, LED2 shows a similar CCT of 4021 K and chromaticity coordinates of (0.3835, 0.3910), but the *R*_a_ can be enhanced to 95.2, indicating the great application potential of the device in full-spectrum lighting for enhanced color rendition. The emission spectra and the variation in chromaticity coordinates of the white LED devices under various drive currents are shown in Supplementary Fig. S5, Supplementary Table S4, and Supplementary Table S5. The provided photoelectric parameters also show that the chromaticity coordinates of LED1 shift more than those of LED2, indicating that LED2 has better color stability when the drive current increases from 20 to 120 mA.

## Discussion

The comparison of the emission spectra of LED1 and LED2 (Fig. 4c) reveals three main features: (1) an additional peak appears at ~490 nm in the spectrum of LED2, and it truly compensates for the cyan gap between 470 nm and 500 nm; (2) this cyan-emitting spectrum shows a decrease in the intensity of the two peaks at 455 nm and 550 nm, indicating the noticeable increase in absorption of the blue part in the range of 380–600 nm; and (3) narrow high-intensity peaks in the range of 600–650 nm stay almost unchanged, causing the relative intensity of the red part to increase compared with that of the blue part. The spectral evolution characteristics are complex, and then it was decided to calculate the difference between the spectra to predict the theoretical maximum of *R*_a_. Herein, we define the following relationship: DIFF(λ) = LED2(λ) – LED1(λ), where LED2(λ) is the experimental spectrum of LED2 and LED1(λ) is the experimental spectrum of LED1. The different theoretical spectrum THEOR(λ) can therefore be presented as the sum of spectrum LED(λ) without sample and different additions of DIFF(λ) with scaling coefficient *x*: THEOR(λ) = LED1(λ) + *x* × DIFF(λ). It should be noted that *x* = 0 means a pure LED1(λ) spectrum and *x* = 1 is a pure LED2(λ) spectrum. Several theoretical spectra were obtained by varying *x* in the range of *x* = 0–2 with a step size of 0.1. The *R*_a_ index of each spectrum was calculated, and the dependence of *R*_a_ on *x* was plotted (Fig. 4d). The maximum in the *R*_a_(*x*) function is *x* = 0.9 with *R*_a_(max) = 95.7. Therefore, the *R*_a_ of a LED with cyan phosphor NKLSO:Eu^2+^ can be raised to 95.7, and the fabricated LED2 with *R*_a_ = 95.2 is close to this maximum (Fig. 4d). The spectrum with this theoretical maximum was calculated using the equation of THEOR(λ) and *x* = 0.9, and it also showed similarity with the spectrum of LED2 (Fig. 4c), which indicates that the theoretical prediction is reasonable. However, the simulation of optimal peak emission and optimal emission band width is far from reality. Accordingly, by determining the optimal peak emission wavelength in combination with the optimal emission band width of a special cyan-emitting phosphor, the idea mentioned above can be used in future work, especially for similar compounds, by revealing the mechanism that improves the *R*_a_ value.

In summary, we have identified a novel ultra-narrow-band cyan-emitting NKLSO:Eu^2+^ phosphor with *λ*_em_ = 486 nm, an FWHM of only 20.7 nm and a Stokes shift of 1069 cm^−1^. The unprecedented ultra-narrow-band cyan emission and extremely small Stokes shift are attributed to the highly condensed, rigid framework and high cubic symmetry sites for the activator (Eu^2+^). The cyan-emitting phosphor also shows good thermal quenching properties with an integrated emission intensity of 93% at 150 °C, demonstrating that NKLSO:Eu^2+^ is a promising phosphor for enhancing the color-rendering index of pc-LEDs in full-spectrum lighting applications. By combining this newly discovered cyan phosphor NKLSO:8%Eu^2+^, the commercial yellow phosphor YAG:Ce and the commercial red phosphor KSF:Mn^4+^ with a blue InGaN LED chip, we fabricated a warm white LED with an *R*_a_ of 95.2. Compared with that of the white LED without the NKLSO:Eu^2+^, the *R*_a_ has been improved significantly, indicating that the phosphor can compensate for the cyan gap and demonstrates great potential for high-CRI white LED devices.

## Materials and methods

### Synthesis

The designed samples of NKLSO:*x*Eu^2+^ (*x* = 0–15%) were synthesized using the traditional solid-state reaction. Stoichiometric amounts of Na_2_CO_3_ (A.R., Aladdin), K_2_CO_3_ (A.R., Aladdin), Li_2_CO_3_ (A.R., Aladdin), SiO_2_ (A.R., Aladdin), and Eu_2_O_3_ (99.99%, Aladdin) were homogeneously mixed and ground with ethanol for half an hour. The mixtures were first sintered at 550 °C for 5 h in air and then sintered three times at 750 °C for 4 h under a reducing atmosphere of N_2_-H_2_ (10%) in a tube furnace. The obtained samples were cooled to RT and were reground into fine powders for further characterization.

### Characterization

The powder X-ray diffraction (XRD) data of NKLSO:*x*Eu^2+^ for Rietveld analysis were collected at RT by a Bruker D8 ADVANCE powder diffractometer, operating at 40 kV and 40 mA with monochromatized Cu Kα radiation (*λ* = 1.5406 Å). Rietveld refinement was performed using TOPAS 4.2. A ^7^Li solid-state NMR spectrum was obtained on a JNM-ECZ600R instrument at 15 kHz, and the external reference was LiCl. The morphology of the powder sample was observed by SEM (SEM, JEOL JSM-6510). The elemental composition and elemental mapping were obtained using energy dispersive EDS that was attached to the SEM. The diffuse reflectance spectra at RT were measured on a Hitachi UH4150 ultraviolet-visible-near infrared spectrophotometer with white BaSO_4_ for calibration. The PL and PLE spectra at RT were recorded by an Edinburgh FLS920 fluorescence spectrophotometer with a Xe900 lamp as the excitation source. The internal/external quantum efficiency values were measured using the integrating sphere on the same FLS920 instrument, and white BaSO_4_ powder was used as a reference to measure the absorption. The luminescence decay curves were measured by an FLS920 instrument using an nF900 flash lamp as the excitation source. The low-temperature (80 K) measurements were performed using the FLS920 with an Oxford Instrument, and the sample was cooled by liquid nitrogen. The temperature-dependent spectra were obtained by a Hitachi F-4600 fluorescence spectrophotometer with a heating apparatus as the heating source and a 150 W Xe lamp as the excitation source.

### WLED fabrication

White LEDs were fabricated with the cyan phosphor NKLSO:8%Eu^2+^, the commercial yellow phosphor YAG:Ce, the commercial red phosphor KSF:Mn^4+^ and a blue LED InGaN chip (*λ* = 455 nm). The phosphors were thoroughly mixed with epoxy resin, and the obtained phosphor−epoxy resin mixture was coated on the LED chips. The photoelectric properties, including the emission spectra, CCT, color-rendering index (CRI, *R*_a_), luminous efficacy, and CIE color coordinates of the LEDs, were measured by using an integrating sphere spectroradiometer system (ATA-1000, Ever fine).

## Supplementary information

Supplementary-CIF file of Na_0.5_K_0.5_Li_3_SiO_4_

Supplementary Material
