# Supplementary material for: Emerging ultra-narrow-band cyan-emitting phosphor for white LEDs with enhanced color rendition
Source: Light Sci Appl. 2019 Apr 10;8:38. doi: 10.1038/s41377-019-0148-8 (PMC6456736; doi:10.1038/s41377-019-0148-8)
Supplement: Supplementary file 2 — Supplementary Material [file 41377_2019_148_MOESM2_ESM.docx]

**Supplementary Information**

Emerging Ultra-Narrow-Band Cyan-Emitting Phosphor for White LEDs with Enhanced Color Rendition

Ming Zhao^1^, Hongxu Liao^1^, Maxim S. Molokeev^2,3,4^, Yayun Zhou^5^, Qinyuan Zhang^5^, Quanlin Liu^1^ and Zhiguo Xia^1,5 *^

^1^The Beijing Municipal Key Laboratory of New Energy Materials and Technologies, School of Materials Sciences and Engineering, University of Science and Technology Beijing, Beijing 100083, China

^2^Laboratory of Crystal Physics, Kirensky Institute of Physics, Federal Research Center KSC SB RAS, Krasnoyarsk 660036, Russia

^3^Siberian Federal University, Krasnoyarsk, 660041, Russia

^4^Department of Physics, Far Eastern State Transport University, Khabarovsk, 680021, Russia

^5^State Key Laboratory of Luminescent Materials and Devices and Guangdong Provincial Key Laboratory of Fiber Laser Materials and Applied Techniques, South China University of Technology, Guangzhou 510641, China

Corresponding Author

* E-mail: [xiazg@ustb.edu.cn](mailto:xiazg@ustb.edu.cn)

**
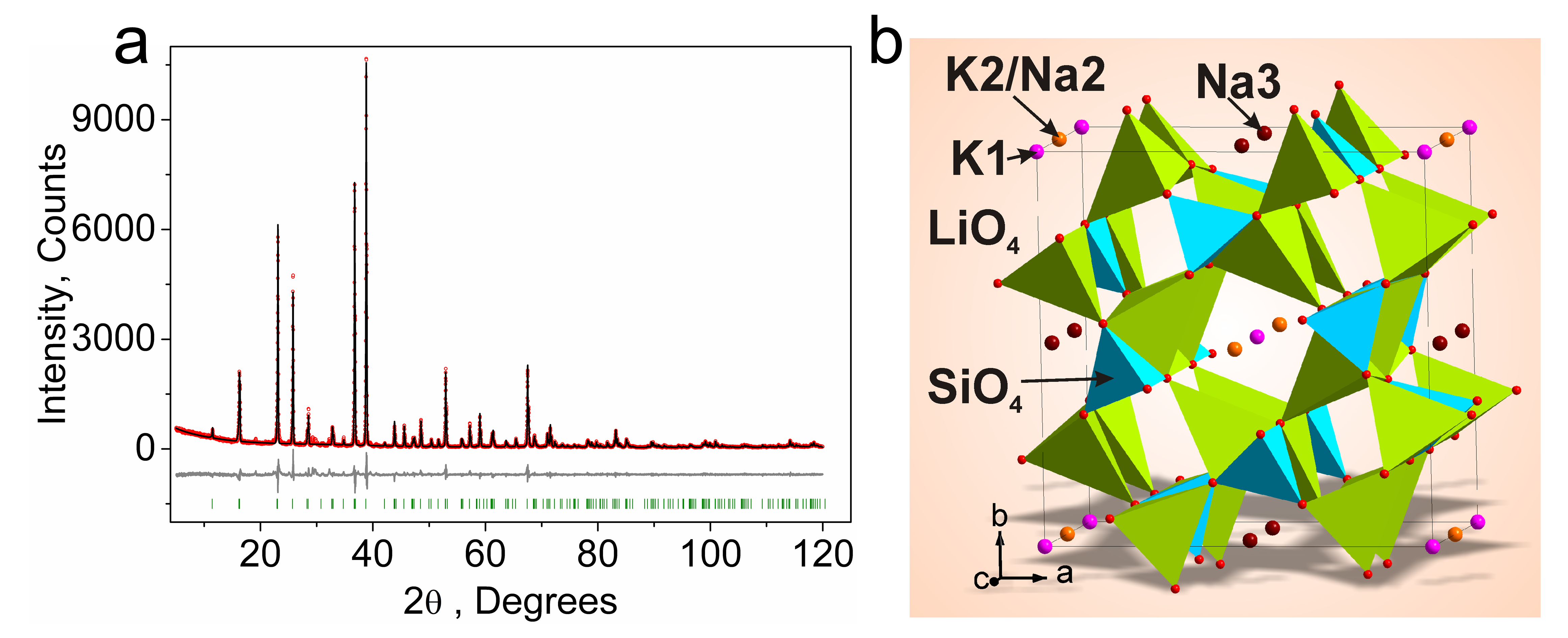
**

**Figure S1 a** The experimental, calculated, and difference refinement XRD pattern of Na_0.5_K_0.5_Li_3_SiO_4_. **b** Crystal structure of Na_0.5_K_0.5_Li_3_SiO_4_.





**Figure S2** The diffuse reflectance spectra of NKLSO:*x*Eu^2+^(*x* = 1-15%).


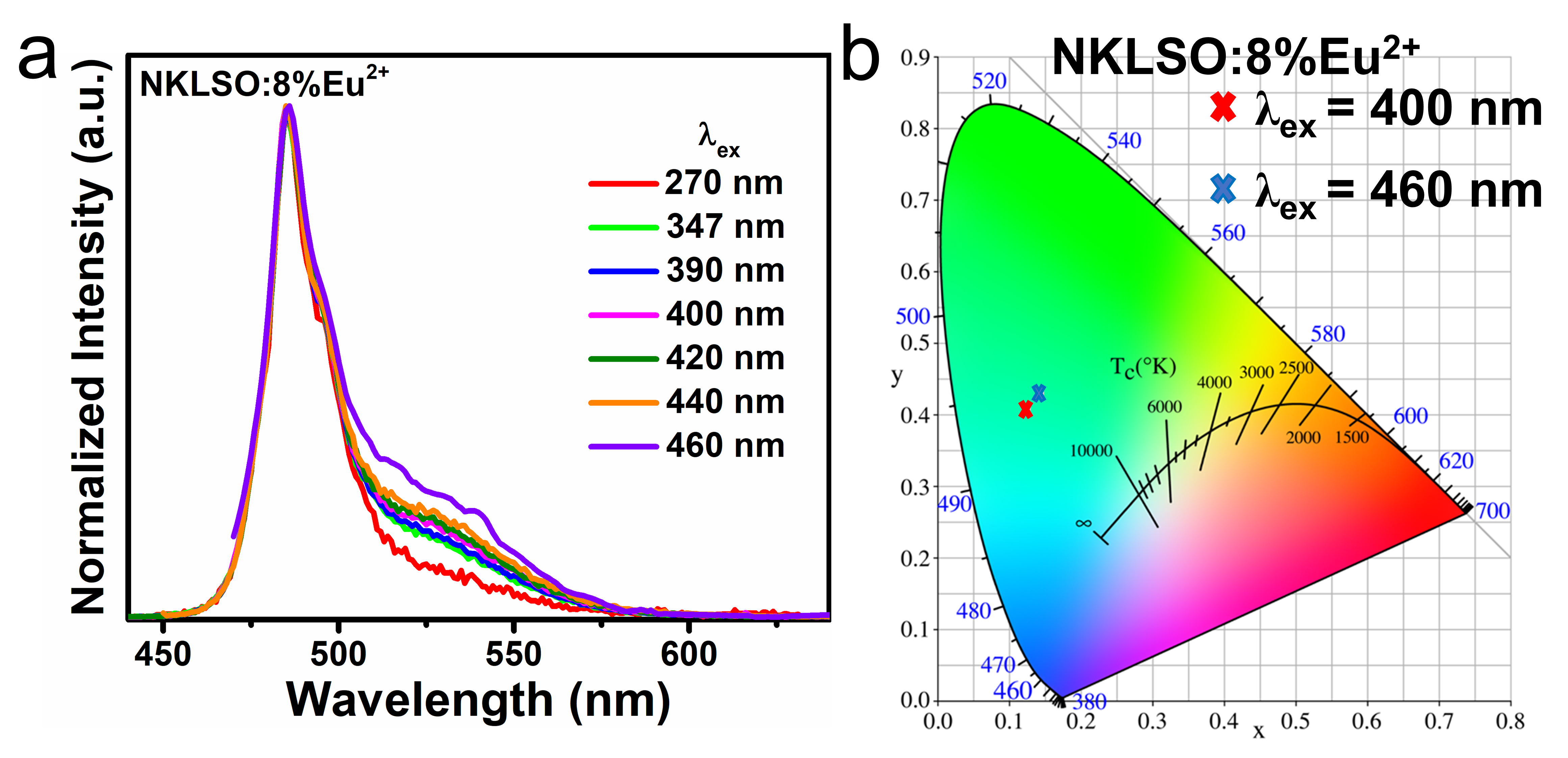


**Figure S3 a** The normalized PL spectra of NKLSO:8%Eu^2+^ under different excitation wavelengths. **b** CIE 1931 color coordinates of the emission spectra under 400 nm and 460 nm excitation of NKLSO:8%Eu^2+^.





**Figure S4** Excitation lines of BaSO_4_ and NKLSO:8%Eu^2+^, and the emission spectrum of NKLSO:8%Eu^2+^ collected by using an integrating sphere. The inset shows a magnification of the emission spectrum of NKLSO:8%Eu^2+^.

^#^ Note: In our work, the external and internal quantum efficiency were measured using the integrated sphere on FLS920 instrument, and white BaSO_4_ powder was used as a reference to measure the absorption. The external (*η*_0_), internal (*η*_i_) quantum efficiencies (QEs) and absorption efficiency (*α*_abs_) were calculated by using the following equations, ^1, 2^

$\eta_{0}\text{=}\frac{\varepsilon}{\delta}\text{=}\frac{\int L_{S}}{\int E_{R}}$, $\eta_{\text{i}}\text{=}\frac{\varepsilon}{\alpha}\text{=}\frac{\int L_{S}}{\int E_{R}-\int E_{S}}$ , $\alpha_{\text{abs}}\text{=}\frac{\alpha}{\delta}\text{=}\frac{\int E_{R}-\int E_{S}}{\int E_{R}}$

where *ε* is the number of photons emitted by the sample, *δ* is the number of total photons excited by the light source and *α* is the number of photons absorbed by the sample. *L_S_* is the luminescence emission spectrum of the sample; *E_R_* is the spectrum of the excitation light with BaSO_4_ in the sphere; *E_S_* is the spectrum of the excitation light with the sample in the sphere; and all the spectra were collected using the sphere.


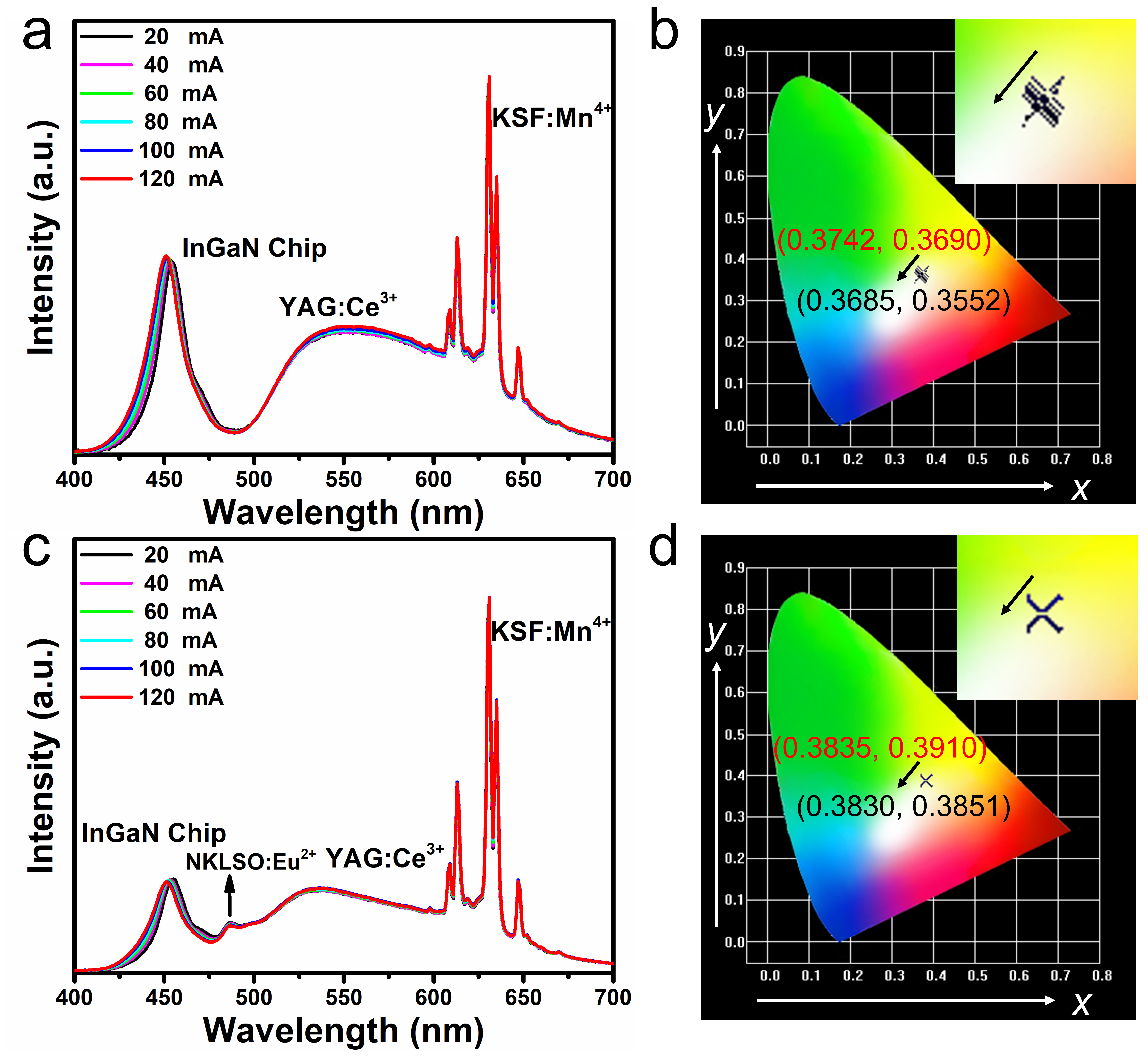


**Figure S5** Emission spectra of the white LED devices fabricated with the commercial yellow phosphor YAG:Ce , the commercial red phosphor KSF:Mn^4+^ and without (**a**) or with (**c**) the cyan phosphor NKLSO:8%Eu^2+^ on a blue LED InGaN chip (λ = 455 nm) under various current. (**b**), (**d**) Current-dependent color coordinates variation of the two fabricated white LEDs.

**Table S1** Main parameters of processing and refinement of the Na_0.5_K_0.5_Li_3_SiO_4_ sample.

| Compound | Na_0.5_K_0.5_Li_3_SiO_4_ |
| --- | --- |
| Sp.Gr. | *I*4/*m* |
| *a*, Å | 10.9447 (1) |
| *c*, Å | 6.26244 (8) |
| *V*, Å^3^ | 750.16 (2) |
| *Z* | 8 |
| *2θ*-interval, º | 5-120 |
| *R_wp_*, % | 12.88 |
| *R_p_*, % | 9.20 |
| *R_exp_*, % | 7.39 |
| *χ^2^* | 1.74 |
| *R_B_*, % | 3.19 |

**Table S2** Fractional atomic coordinates and isotropic displacement parameters (Å^2^) of Na_0.5_K_0.5_Li_3_SiO_4_.

| Atom | Wyck | *x* | *y* | *z* | *B*_iso_ | Occ. |
| --- | --- | --- | --- | --- | --- | --- |
| Si1 | 8h | 0.2187 (2) | 0.4234 (3) | 0 | 0.8 (1) | 1 |
| K1 | 2a | 0 | 0 | 0 | 1.5 (2) | 1 |
| K2 | 2b | 0 | 0 | 0.5 | 1.0 (2) | 0.67 (2) |
| Na2 | 2b | 0 | 0 | 0.5 | 1.0 (2) | 0.33 (2) |
| Na3 | 4d | 0.5 | 0 | 0.25 | 2.0 (2) | 1 |
| Li1 | 16i | 0.7305 (10) | 0.3945 (11) | 0.251 (2) | 1.0 (2) | 1 |
| Li2 | 8h | 0.7152 (15) | 0.9256 (18) | 0 | 1.0 (2) | 1 |
| O1 | 8h | 0.4360 (6) | 0.1617 (5) | 0 | 1.0 (1) | 1 |
| O2 | 8h | 0.8928 (5) | 0.6660 (5) | 0 | 1.0 (1) | 1 |
| O3 | 16i | 0.9124 (3) | 0.2019 (4) | 0.2817 (6) | 1.0 (1) | 1 |

**Table S3** Main bond lengths (Å) of Na_0.5_K_0.5_Li_3_SiO_4_.

| K1—O3 | 2.985 (4) | Li1—O1 | 2.02 (1) |
| --- | --- | --- | --- |
| K2—O3 | 2.770 (4) | Li1—O2 | 1.93 (1) |
| Na2—O3 | 2.770 (4) | Li1—O3 | 2.91 (1) |
| Na3—O1 | 2.464 (5) | Li1—O3 | 1.90 (1) |
| Na3—O2 | 2.670 (5) | Li1—O3 | 2.15 (1) |
| Si1—O1 | 1.660 (7) | Li2—O1 | 1.91 (2) |
| Si1—O2 | 1.564 (6) | Li2—O2 | 2.06 (2) |
| Si1—O3 | 1.625 (4) | Li2—O3 | 1.98 (1) |

**Table S4** The photoelectric properties of the white LED1 fabricated using the commercial yellow phosphor YAG:Ce and the commercial red phosphor KSF:Mn^4+^ with a blue LED InGaN chip (λ = 455 nm) excitation under various drive currents.

| Current(mA) | CIE *x* | CIE *y* | *R_a_* | CCT | luminous efficacy (lm/W) |
| --- | --- | --- | --- | --- | --- |
| 20 | 0.3742 | 0.3690 | 86 | 4119 | 174.98 |
| 40 | 0.3722 | 0.3647 | 85.8 | 4146 | 159.39 |
| 60 | 0.3711 | 0.3615 | 85.5 | 4156 | 147.2 |
| 80 | 0.3703 | 0.3593 | 85.3 | 4163 | 136.48 |
| 100 | 0.3694 | 0.3573 | 85 | 4179 | 127.71 |
| 120 | 0.3685 | 0.3552 | 84.9 | 4193 | 120.12 |

**Table S5** The photoelectric properties of the white LED2 fabricated using the cyan phosphor NKLSO:8%Eu^2+^, the commercial yellow phosphor YAG:Ce and the commercial red phosphor KSF:Mn^4+^ with a blue LED InGaN chip (λ = 455 nm) excitation under various drive currents.

| Current(mA) | CIE *x* | CIE *y* | *R_a_* | CCT | luminous efficacy (lm/W) |
| --- | --- | --- | --- | --- | --- |
| 20 | 0.3835 | 0.3910 | 95.2 | 4021 | 119.92 |
| 40 | 0.3833 | 0.3889 | 94.7 | 4012 | 111.72 |
| 60 | 0.3835 | 0.3878 | 94.4 | 3998 | 104.85 |
| 80 | 0.3838 | 0.3873 | 94.1 | 3988 | 98.94 |
| 100 | 0.3840 | 0.3868 | 93.9 | 3978 | 93.54 |
| 120 | 0.3830 | 0.3851 | 93.6 | 3993 | 88.55 |

Ref.:

1. Wang, L.*, et al.* Highly efficient narrow-band green and red phosphors enabling wider color-gamut LED backlight for more brilliant displays. *Opt. Express* **23**, 28707-28717 (2015).

2. Ohkubo, K. & Shigeta, T. Absolute fluorescent quantum efficiency of NBS phosphor standard samples. *J. Illum. Eng. Inst. Japan* **83**, 87-93 (1999).
